# SUMOylation of sPRDM16 promotes the progression of acute myeloid leukemia

**DOI:** 10.1186/s12885-015-1844-2

**Published:** 2015-11-11

**Authors:** Song Dong, Jieping Chen

**Affiliations:** Department of Hematology, Southwest Hospital, Third Military Medical University, 30 Gaotanyan Street, Chongqing, 400038 People’s Republic of China

**Keywords:** SUMOylation, sPRDM16, acute myeloid leukemia

## Abstract

**Background:**

In addition to genetic and epigenetic alteration, post-translational modification of proteins plays a critical role in the initiation, progression and maturation of acute myeloid leukemia (AML).

**Methods:**

The SUMOylation site of sPRDM16 at K568 was mutated to arginine by site-directed mutagenesis. THP-1 acute myeloid leukemia cells were transduced with a lentivirus containing wild type or K568 mutant sPRDM16. Proliferation, self-renewal and differentiation of transduced THP-1 cells were analyzed both *in vitro* cell culture and in mouse xenografts. Gene expression profiles were analyzed by RNA-seq.

**Results:**

Overexpression of sPRDM16 promoted proliferation, enhanced self-renewal capacity, but inhibited differentiation of THP-1 acute myeloid leukemia cells. We further confirmed that K568 is a bona fide SUMOylation site on sPRDM16. Mutation of the sPRDM16 SUMOylation site at K568 partially abolished the capacity of sPRDM16 to promote proliferation and inhibit differentiation of acute myeloid leukemia cells both *in vitro* and in mouse xenografts. Furthermore, THP-1 cells overexpressing sPRDM16-K568R mutant exhibited a distinct gene expression profile from wild type sPRDM16 following incubation with PMA.

**Conclusions:**

Our results suggest that K568 SUMOylation of sPRDM16 plays an important role in the progression of acute myeloid leukemia.

## Background

Acute myeloid leukemia (AML) is a serious disease of the hematopoietic system characterized by de-differentiation and uncontrolled proliferation of immature hematopoietic precursor cells in the bone marrow [1]. Leukemia results from the accumulation of genetic and epigenetic alterations during the multistep process of tumorigenesis, including activation of oncogenes and/or inactivation of tumor suppressor genes. Many transcription factors have been shown to play an important role in aggressive hematological tumors. Within the past decade evidence that post-translational modification of proteins, including phosphorylation [2], acetylation [3], ubiquitination [4] and SUMOylation [5, 6], plays a critical role in the initiation, progression and maturation of AML has accumulated.

Positive regulatory domain I-binding factor 1 and retinoblastoma-interacting zinc finger protein-1 (PRDM16) is a transcription factor [7]. PRDM16 is also termed MEL1 because it shares 63 % sequence similarity with PRDM3/MECOM (MDS1 and EVI1 complex) [8]. PRDM16 encodes two isoforms: the full-length PRDM16 (or MEL1) and the short-form sPRDM16 (or MEL1S) [9]. PRDM16, but not sPRDM16, contains a 134-amino acid PR domain, which is highly homologous to the SET domain, a structural hallmark of histone methyltransferases [7, 10]. PRDM16 plays an important role in palatogenesis [11], maintenance of hematopoietic [12] and neuronal stem cells [13], and adipose tissue differentiation [14–17]. It acts as an H3K9me1 methyltransferase, which is required to maintain the integrity of mammalian heterochromatin [7, 9, 10, 18]. PRDM16 is reported to contribute to translocation-induced leukemia [7, 9, 10, 18]. However, only the PR domain-negative isoform of sPRDM16 is potentially oncogenic in leukemia [10], and the underlying molecular mechanisms are largely unknown.

Small ubiquitin-related modifier (SUMO) is a highly conserved ubiquitin-like protein which acts as a reversible and highly dynamic post-translational modifier of a large number of proteins [19, 20]. SUMOylation is catalyzed by a multistep enzymatic cascade including activating (E1), conjugating (E2), and ligating (E3) enzymes. SUMOylation alters the localization, activity or stability of target proteins [19], and is reversed by a family of Sentrin/SUMO-specific proteases (SENPs).

Accumulating evidence has shown that SUMOylation plays a wide range of roles in the regulation of growth and development of all eukaryotes, for example, influencing transcriptional regulation and genome integrity. Deregulation of SUMOylation has been found in many human diseases including cancer, seizures and Alzheimer’s diseases [21]. Recently, sPRDM16 was reported to be SUMOylated [22], and we have found PRDM16 to also be SUMOylated (data not shown). However, the role of PRDM16 SUMOylation in progression of AML is unknown.

In this study, we have explored sPRDM16 SUMOylation in AML *in vivo* and *in vitro*. We found that SUMOylation of sPRDM16 regulated expression of genes during AML differentiation, and promoted AML progression while inhibiting differentiation of AML cells.

## Methods

### Plasmids and antibodies

The plasmid MSCV-PRDM16 was purchased from Addgene (Cambridge, MA, USA). Plasmid FLAG-sPRDM16 was generated from MSCV-PRDM16 by standard molecular cloning methods. Plasmids HA-SUMO1, HA-UBC9, RGS-SENP1 and RGS-SENP1-mutant were designed and developed as previously described [23]. Anti-Flag antibody was obtained from Sigma (clone M2, St. Louis, MO, USA); anti-HA antibody was purchased from COVANCE (Beijng, China); anti-RGS antibody was obtained from QIAGEN (Germantonw, MD, USA); and anti-SUMO1 and PRDM16 antibodies from Abcam (Cambridge, MA, USA).

### Site-directed mutagenesis

The potential SUMOylation residues from lysine (K) to arginine (R) in sPRDM16 were mutated using the QuickChange^TM^ site-directed mutagenesis kit (Stratagene, La Jolla, CA, USA).

FLAG-sPRDM16-K568R was generated with the following primers:

Sense 5′-TTGCTGGTCAGGGCTGAGCCA -3′ and Antisense 5′-TGGCTCAGCCCTGACCAGCAA-3′.

### Cell culture

HEK293T cells were cultured in DMEM (Hyclone) supplemented with 10 % fetal bovine serum (Gibco) and 1 % penicillin-streptomycin (Gibco). THP-1 cells were maintained at 37 °C with 5 %CO_2_ in RPMI-1640 Medium (Hyclone) supplemented with 0.05 mM 2-mercaptoethanol (Sigma) and 10 % fetal bovine serum (Gibco). Plasmids were transiently transfected into HEK293T cells using Lipofectamine^TM^2000 (Invitrogen) according to manufacturer’s instructions.

### Evaluation of cell adherence (morphological differentiation)

Differentiation of THP-1 cells to macrophage-like cells was assessed by measureing adherence to plastic cell culture wells. Log phase cells were centrifuged and resuspended at at 1 × l0^6^ cell/ml in fresh RPMI1640 complete medium containing 3 nM PMA and incubated for 24 h. Nonadherent cells were collected from the supernatant after washing, then adherent cells were separated gently by cell scraper on ice. The adherent and non-adherent cells were counted, and the sum correlated well with the original number of cells plated. To evaluate of differentiation, control and treated cells were removed from the Petri dishes, pelleted by centrifugation, and resuspended in 1 ml fresh medium.

### Western blot

Total protein was extracted from cells or tissues using radioimmune precipitation assay (RIPA) buffer (50 mM Tris-HCl pH7.4, 150 mM NaCl, 1 % NP-40, 0.1 % SDS, 1 mM EDTA) with 1 % protease inhibitor cocktail. Equal amounts of protein extracts (40 μg) were separated by 10 % sodium dodecyl sulfate-polyacrylamide gel electrophoresis (SDS-PAGE) and transferred onto a PVDF membrane. Membranes were blocked with 5 % w/v non-fat dry milk dissolved in Tris-buffered saline plus Tween-20 (TBS-T; 0.1 % Tween-20; pH 8.3) at room temperature for 1 h, and incubated with primary antibodies at 4 °C overnight. After washing with TBS-T, membranes were incubated with horseradish peroxidase (HRP)-labeled secondary antibodies for 1 h at room temperature. Immunobands were visualized using enhanced chemiluminescence (ECL) kit (GE Healthcare, Waukesha, WI, USA) according to manufacturer’s instructions and exposed to X-ray films.

### Immunoprecipitation

Cells were collected at 48 h after transfection and lysed using an ice-cold RIPA buffer (50 mM Tris-HCl pH7.4, 150 mM NaCl, 1 % NP-40, 0.1 % SDS, 1 mM EDTA) with 10 mM N-ethylmaleimide, 1 mM PMSF and protease inhibitors (Roche). Cell lysis was performed on ice for 20 min and the cell lysate was sonicated for 5 s three times. After centrifugation at 14,000 g for 10 min at 4 °C, the supernatants were added to the appropriate antibody coupled to 20 μl of anti-FLAG M2 agarose beads (Sigma). The bead suspension was incubated at 4 °C for 2 h on a rotating shaker. Beads were then washed 5 times with RIPA buffer, mixed with 15 μL 2× SDS sample buffer and boiled at 100 °C for 5 min. The samples were subjected to Western blot analysis.

### Lentiviral transduction

To obtain lentivirus particles, 5 × 10^5^ HEK293T packaging cells were plated in 60-mm culture dishes and transiently transfected with 2 μg of each lentivirus vector mixture (pCDH-sPRDM16 1 μg, psPAX2 0.75 μg, pMD2.G 0.25 μg) together with 5 μl Lipofectamine 2000 (Invitrogen). Supernatant containing lentivirus was collected 36 h after transfection, filtered using 0.45-mm filters and used immediately for infection. Logarithmically growing THP-1 cells were transduced with lentivirus as previously described [24].

### Cell proliferation assays

To assess cellular proliferation, cells were seeded in 24-well plates (2 × 10^5^ cells in 1 ml medium per well) and counted each day using a hematocytometer and trypan blue staining to exclude dead cells. Colorimetric proliferation assays were performed in 96-well plates as 8-fold measurements. A WST-8 [2-(2-methoxy-4-nitrophenyl)-3-(4-nitrophenyl)-5-(2,4-disulfophenyl)-2H-tetrazolium, monosodium salt] assay (Dojindo, Shanghai, China) was conducted using 2000 cells in 100 μl medium per well in 96-well plates. The cell counting kit-8 (CCK-8) assay was performed according to the manufacturers’ recommendations.

### Soft agar colony formation assay

A soft agar suspension (0.35 % agar) containing colony-forming cells was plated over a soft agar underlay (0.6 % agar). THP-1 cell complete culture medium was added and changed twice a week. After 1000 cells were embedded in the soft agar in 6-well plates (end concentration 0.35 % agar in complete medium) and grown over 12 days, colony numbers were counted under microscope.

### Flow cytometry

For flow cytometry, cells were fixed with 4 % paraformaldehyde in PBS for 10 min and then blocked with 1 % BSA in PBS for 10 min at room temperature. Fixed, blocked cells were incubated with APC-conjugated anti-CD11b monoclonal antibody (BD PharMingen) for 15 min on ice, and cells were washed 3 times with 1 % BSA in PBS. Staining was analyzed using a FACS Calibur instrument (BD PharMingen).

### Animal systemic leukemia models

NOD.CB17-Prkdc^scid^/J (NOD/SCID) mice were purchased from Shanghai SLAC laboratory Animal Co., Ltd. (Shanghai, China). Animals were maintained at the animal facility of Shanghai Jiao Tong University School of Medicine in accordance with the local regulations and handled under sterile conditions. The study protocol was approved by the Review Committee for the Use of Human or Animal Subjects of Shanghai Jiao Tong University School of Medicine. Transplantations were performed by intravenous injections of six to eight week old mice. THP-1 cells (1 × 10^7^ cells per mouse in 200 ml saline vehicle) were injected into the tail vein to create the leukemia models 5 h after total body irradiation with 1.5 Gy using a 137Cs source to enhance angiogenic potential [25]. After transplantation, the mice were monitored for leukemia symptoms, such as weight loss, hunch-back, and decreased activity. All procedures were carried in accordance with national and international laws and policies.

### MRNA-sequencing and data analysis

RNAs from the THP-1 cell line with stable expression of sPRDM16-WT or sPRDM16–K568R or a mock-transfected cell line were purified using Trizol^TM^ method and subsequently cleaned using RNAeasy Kit (Qiagen). The polyadenylated RNAs purified from the cells were used for the construction of a sequencing library using the ScriptSeq Complete Gold Kit (Epicentre, Illumina). Cluster generation and sequencing were carried out using standard procedures in Hi-Seq 2500 Illumina platform. We used a single-end sequencing protocol to generate a 50 nt read at each end. RNA-seq reads were aligned to the human genome using TopHat (Johns Hopkins University, Baltimore, MD, USA). Cufflinks was employed to normalize Data and perform relevant comparisons among the different samples. Gene ontology analysis was performed using DAVID GO analysis software to search for enriched pathways.

### Quantitative real-time PCR analysis

Total RNA was extracted by Trizol kit (Invitrogen) and treated with DNase (Promega). Complementary DNA was reverse transcribed using M-MLV reverse transcriptase and random hexamers according to the manufacturer’s protocol (Takara). All experiments were performed with Power SYBR® Green PCR Master Mix (Applied Biosystems) using the LightCycler® 480 Real-Time PCR System (Roche). PCR was carried out in triplicate and standard deviations representing experimental errors were calculated. Differences in cDNA input were normalized to GAPDH. All data were analyzed by the LightCycler® 480 software (Roche). The following PCR primers were used:

hKLF10-forward-5′-ACTGCGGAGGAAAGAATGGA-3′, hKLF10-reverse-5′-CTGGGAGGAGTGCTGGGAAC-3′; hCCL5-forward-5′-GCTGTCATCCTCATTGCTAC-3′, hCCL5-reverse-5′-CATTTCTTCTCTGGGTTGGC-3′; IL6R-forward-5′-TGCCAGTATTCCCAGGAGTC-3′, IL6R-reverse-5′-GGCAGTGACTGTGATGTTGG-3′; hLIF-forward-5′-ACAGAGCCTTTGCGTGAAAC-3′, hLIF-reverse-5′-TGGTCCACACCAGCAGATAA-3′; hNUMB-forward-5′-CGATGACCAAACCAGTGACAG-3′, hNUMB-reverse-5′-AGAGGGAGTACGTCTATGACCG-3′; hBCL3-forward-5′-ACTGCCTTTGTACCCCACTC-3′, hBCL3-reverse-5′-GGTATAGGGGTGTAGGCAGGT-3′; hHDAC9-forward-5′-GGATCAAAGCTCTCCACCCC-3′, hHDAC9-reverse-5′-TGGGCTCAGAGGCAGTTTTT-3′.

GAPDH-forward-5′-AGAAGGCTGGGGCTCATTTG-3′, GAPHD-reverse-5′-AGGGGCCATCCACAGTCTTC-3′.

Results were expressed as relative expression, normalized to the internal control.

### Statistical analysis

All data are presented as mean ± standard deviation (S.D.). Statistical analysis was performed using Student’s t-test and values of *P* ≤ 0.05 were considered statistically significant.

## Results

### sPRDM16 promotes proliferation, enhances self-renewal capacity, while inhibiting differentiation of acute myeloid leukemia cells

**Fig. 1:**
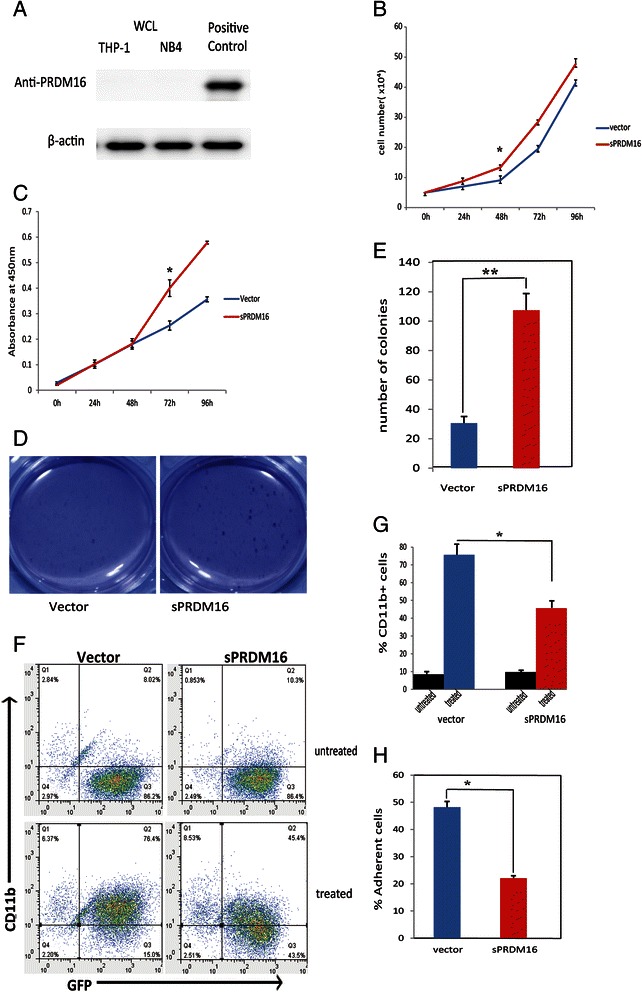
sPRDM16 promoted the proliferation, enhanced the capacity for self-renewal and inhibited the differentiation of acute myeloid leukemia cells. **a** Protein levels of sPRDM16 in THP-1 and NB4 cell lines. The original whole-cell lysates (WCL) were analyzed by immunoblotting (IB) with anti-PRDM16 or anti-actin antibodies. **b** Overexpression of sPRDM16 promoted proliferation of THP-1 cells. THP-1 cells stably transfected with either vector (Vector-THP-1) or sPRDM16 (sPRDM16-WT-THP-1) were cultured and cell number was counted at the indicated time. Data are presented as mean ± S.D. of three independent experiments in RPMI-1640 medium. The number of vector and sPRDM16 differed significantly at 48 h (P < 0.01). **c** Proliferation of the THP-1 cells was determined using CCK-8 assay. The absorbance of the CCK-8 assay solution was detected with plate reader (450 nm filter). Data are presented as means ± S.D. of three independent experiments. Difference between two cell lines was significant at 48 h (*P* < 0.01). **d** Overexpression of sPRDM16 increased colony formation of THP-1 cells. THP-1 cells stably transfected with either vector (Vector-THP-1) or sPRDM16 (sPRDM16-WT-THP-1) were seeded in 1 ml of medium containing 10 % FBS with 0.35 % soft agar at 1 × 10^3^ cells per well and layered onto the base soft agar medium. The photographs were taken after 12 days. Images are representative of three independent experiments. **e** The number of colonies in colony formation assay was scored after 12 days. The colonies were visualized by staining with 0.5 % crystal violet. The experiments were analyzed in triplicate and colonies larger than 50 μm in diameter were counted under microscope. Columns, mean of triplicate experiments; bars, S.D. An unpaired (equal variance) t-test was performed to compare sPRDM16–WT and vector control (* * *P* < 0.01). **f** Overexpression of sPRDM16 suppressed differentiation of THP-1 cells. Logarithmically growing cells were exposed to 3 nM PMA for 24 h. The percentage of cells expressing the monocytic maturation marker CD11b was determined by flow cytometry. The results are representative of three independent experiments. **g** The results of flow cytometry were represented as a histogram. Values represent the mean ± S.D. for experiments performed in triplicate. *P < 0.01 represents a significant difference from the cells treated with PMA (*n* = 3); columns, mean of triplicate experiments; bars, S.D. **h** The same THP-1 cells were treated as E. Logarithmically growing cells were treated with 3 nM PMA for 24 h. The percentage of adherent cells was calculated. Values represent the mean ± S.D. for experiments performed in triplicate. *P < 0.01 represents a significant difference from cells treated with PMA (*n* = 3); columns, mean of triplicate experiments; bars, S.D.

Protein expression of sPRDM16 wasn’t detected in THP-1 or NB4 cell line (Fig. 1a) and very few leukemic cell lines express sPRDM16 [10]. To investigate the role of sPRDM16 in AML progression, we cloned the DNA fragment from the internal initiation codon ATG597 in exon 4 to exon 17 of PRDM16 into the PCDH lentivirus vector with FLAG-tag at the N-terminal, and established stablly infected cell lines. We first monitored the proliferation of THP-1 cells stably transfected with either vector (Vector-THP-1) or sPRDM16 (sPRDM16-WT-THP-1). We found that cells overexpressing sPRDM16 proliferated more rapidly than Vector-THP-1-transfected cells (Fig. 1b). Similar results were obtained by CCK-8 assay (Fig. 1c).

To assess whether sPRDM16 enhanced self-renewal of leukemia cells, we performed a soft agar colony formation assay in the presence of 10 % FBS and monitored cell growth. After 12 days, sPRDM16-WT-THP-1 cells had formed more soft agar colonies than Vector-THP-1 cells (*P* < 0.01) (Fig. 1d and e.

To determine whether sPRDM16 played a role in the maturation of AML cells, we incubated THP-1 cells with 3 nM PMA for 24 h, and measured cell-surface expression of the monocytic maturation marker CD11b using APC-labeled anti-CD11b antibody by flow cytometry (Fig. 1f). Expression of CD11b did not differ significantly between Vector-THP-1 (8.02 %) and sPRDM16-WT-THP-1 (10.3 %) cells. PMA induced expression of CD11b in 45.4 % of sPRDM16-WT-THP-1 cells, while a significant increase in CD11b expression was observed in Vector-THP-1 cells (76.4 %, P < 0.01) (Fig. 1f and g). Further, when cell adherence was monitored, as an indication of monocyte differentiation, sPRDM16-WT-THP-1 cells demonstrated less adherence (Fig. 1h).

These results indicate that sPRDM16 promoted proliferation and enhanced self-renewal capacity, while inhibing cellular differentiation in AML, suggesting that sPRDM16 may be an oncogene.

### K568 is a bona fide SUMOylation site of sPRDM16

**Fig. 2:**
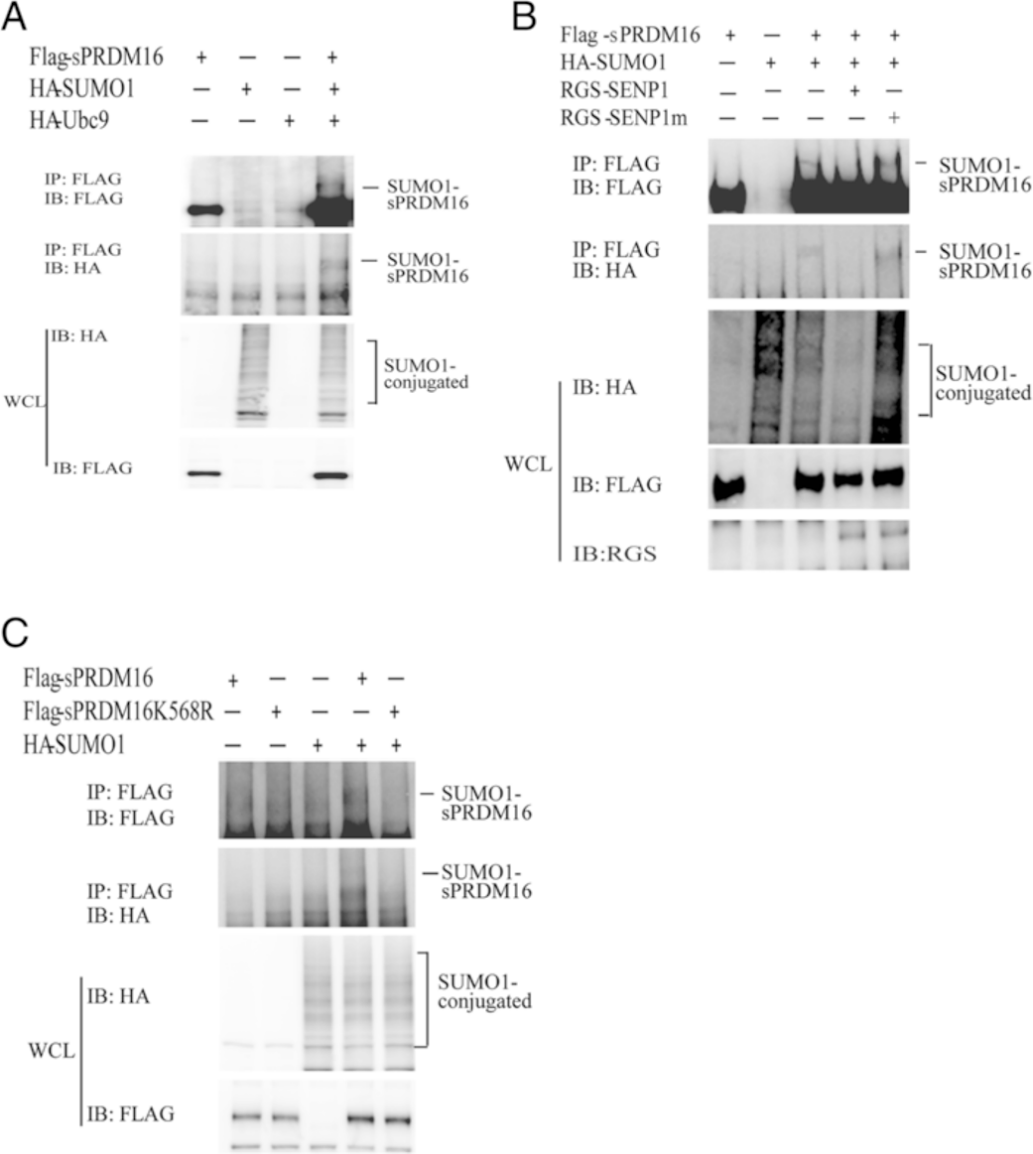
sPRDM16 was SUMOylated by SUMO1 on lysine-568. **a** sPRDM16 was SUMOylated *in vivo*. HEK293T cells were transfected with the indicated plasmids for 36 h. Immunoprecipitation was performed with anti-FLAG M2 agarose beads. The immunoprecipitates (IP) and the original whole-cell lysates (WCL) were analyzed by immunoblotting (IB) with anti-HA or anti-FLAG antibodies. **b** SENP1 de-SUMOylated sPRDM16. HEK293T cells were transfected with HA-SUMO1, Flag-sPRDM16, RGS-SENP1, or RGS-SENP1m as indicated. Flag-sPRDM16 proteins were pulled down by anti-Flag M2 agarose beads from cell lysates. Bound proteins were blotted with anti-Flag. Cell lysate was immunoblotted (IB) with anti-Flag antibody, anti-HA antibody, or anti-RGS antibody. **c** K568 was the primary SUMOylation site of sPRDM16. HEK293T cells were transfected with the indicated plasmids. Cell lysates were immunoprecipitated with anti-FLAG M2 agarose beads, followed by Western blot (WB) analysis using anti-HA or anti-FLAG antibodies

Overexpression of sPRDM16 with loss of p53 induces myeloid leukemia in mice [10]. It was also reported that SUMOylation of sPRDM16 leads to its interaction with CtBP, facilitating the repressor activity of CtBP and blockade of G-CSF-induced myeloid differentiation in L-G3 cells [22]. Thus, we speculated that sPRDM16 SUMOylation may contribute to AML progression. To test this hypothesis, we first confirmed that sPRDM16 was SUMOylated by SUMO1. We co-transfected HEK293T cells with Flag-sPRDM16, HA-SUMO1 and HA-UBC9 plasmids, and performed co-immunoprecipitation assay. As show in Fig. 2a, sPRDM16 was conjugated to SUMO1. Next, we co-expressed Flag-sPRDM16, HA-SUMO1 and SENP1 in HEK293T cells. SUMOylated sPRDM16 was readily detected when Flag-sPRDM16 and HA-SUMO1 were co-transfected. In sharp contrast, the SUMOylated band disappeared when wild type SENP1 was over-expressed, but not when the catalytic mutant SENP1m was expressed (Fig. 2b). To confirm that the SUMO acceptor site of sPRDM16 was K568, we generated sPRDM16 SUMOylation mutant, sPRDM16-K568R and performed SUMOylation assays in HEK293T cells co-transfected with wild-type sPRDM16-WT or mutant sPRDM16-K568R and HA-SUMO1. As expected, one band of SUMOylated Flag-sPRDM16 was observed in the immunoprecipitates of cells transfected with sPRDM16–WT. In contrast, the mutant sPRDM16-K568R completely abolished SUMOylation (Fig. 2c), consistent with the hypothesis that K568 is a bona fide SUMOylation site of sPRDM16 [22].

### SUMOylation contributes to sPRDM16-mediated tumorigenesis in acute myeloid leukemia

**Fig. 3:**
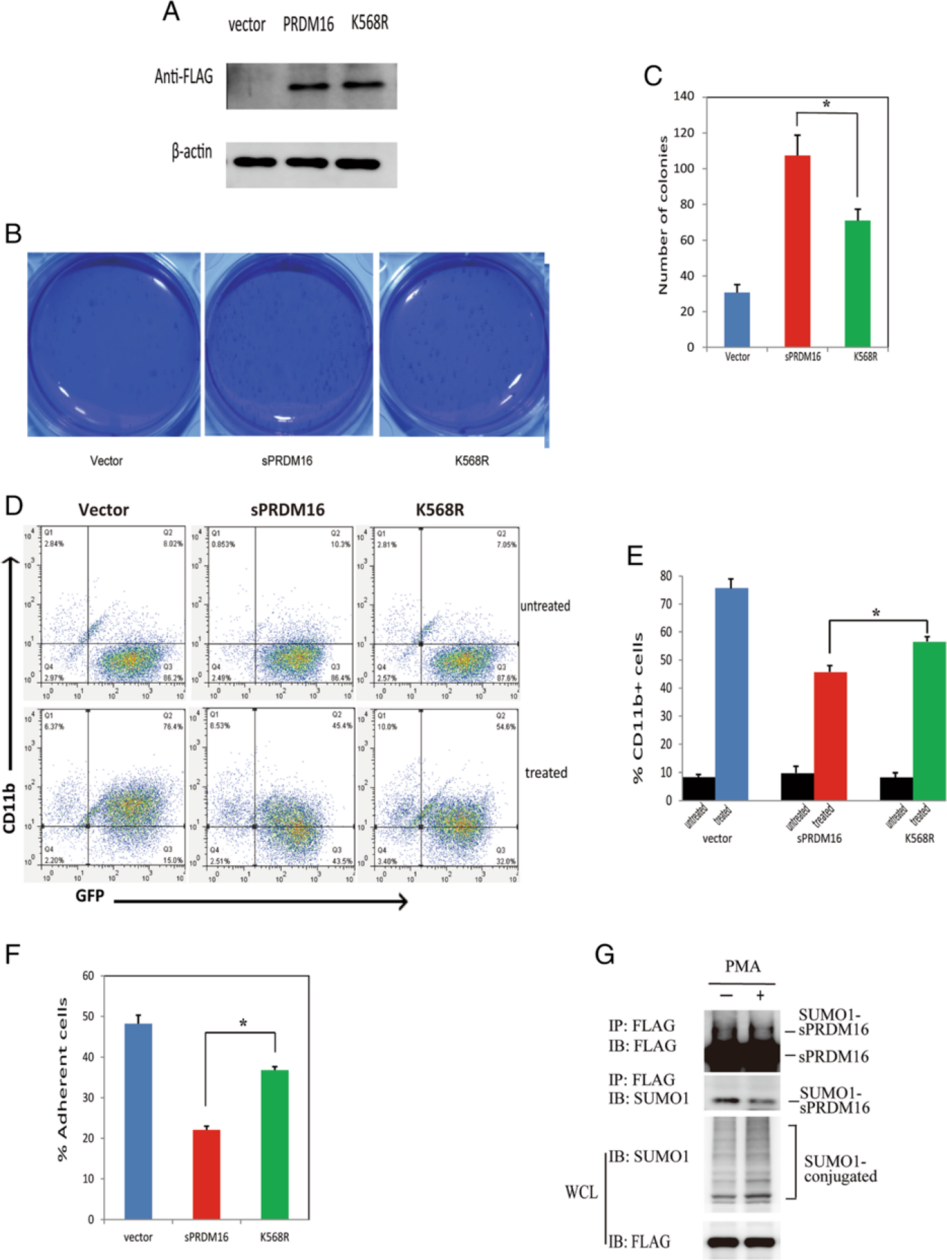
SUMOylation was required for sPRDM16 in the progression of acute myeloid leukemia. **a** Exogenous protein expression of sPRDM16 or K568R in THP-1 cell line. The original whole-cell lysates (WCL) were analyzed by immunoblotting (IB) with anti-FLAG or anti-actin antibodies. **b** THP-1 cells stably transfected with either vector, sPRDM16 or sPRDM16-K568R were seeded in 1 ml of medium containing 10 % FBS with 0.35 % soft agar at 1 × 10^3^ cells per well and layered onto the base soft agar medium. Photographs were taken after 12 days. Images are representative of three independent experiments. **c** The number of colonies of colony formation assay (A) was scored after 12 days. The colonies were visualized by staining with 0.5 % crystal violet. The experiments were analyzed in triplicate and colonies larger than 50 μm in diameter were counted under microscope. Columns, mean of triplicate experiments; bars, S.D. (* * *P* < 0.01). **d** Logarithmically growing THP-1 cells stably transfected with either sPRDM16 or sPRDM16-K568R vector, were exposed to 3 nM PMA for 24 h. The percentage of cells expressing the monocytic maturation marker CD11b was determined by flow cytometry. The dot plot results are representative of three independent experiments. **e** The results of flow cytometry described in Fig. 3C were represented as a histogram. Values represent the mean ± S.D. for experiments performed in triplicate. *P < 0.01 represents a significant difference from the cells treated with PMA (*n* = 3); columns, mean of triplicate experiments; bars, s.d. **f** Logarithmically growing THP-1 cells were treated with 3 nM PMA for 24 h. The percentages of adherence cells on the surface of plastic dish were counted. Values represent the mean ± S.D. for experiments performed in triplicate. *P < 0.01 represents a significant difference from the cells treated with PMA (*n* = 3). Columns, mean of triplicate experiments; bars, S.D. **g** sPRDM16 SUMOylation in THP-1 cells stably expressing sPRDM16-WT was decreased after incubation with PMA. SUMO1-conjugated proteins in THP-1 cells in the presence or absence of PMA for 24 h were immunoprecipitated with anti-FLAG M2 agarose beads. SUMO1-sPRDM16 proteins were blotted with anti-FLAG and anti-SUMO1. Cell lysate was immunoblotted with anti-FLAG and anti-FLAG antibody

To explore the function of sPRDM16 SUMOylation in progression of AML, we generated stable THP-1 cell lines by polyclonal lentiviral infections with Lenti-Vector, sPRDM16-WT or sPRDM16–K568R. Exogenous protein level of sPRDM16 or K568R in THP-1 cell line was shown in (Fig. 3a). We first performed a soft agar colony-forming assay in the presence of 10 % FBS. sPRDM16-WT-THP-1 and sPRDM16-K568R-THP-1 cells formed more numerous and larger colonies than Lenti-Vector transfected cells (Fig. 3b and c). However, colony formation was slower in sPRDM16-K568R transfected cells than sPRDM16-WT-THP-1, suggesting that sPRDM16–K568R reduced the capacity of self-renewal (Fig. 3b and c).

To determine whether SUMOylation of sPRDM16 was involved in differentiation of AML cells, we treated cells stably transfected with Lenti-Vector, sPRDM16–WT and sPRDM16–K568R with 3 nM PMA for 24 h and measured cell surface expression of the monocytic maturation marker CD11b by flow cytometry. Incubation with PMA significantly increased expression of CD11b in Vector-THP-1, sPRDM16-WT and sPRDM16-K568R-THP-1 cells. However, PMA stimulated CD11b expression less potently in sPRDM16-WT-THP-1 cells (45.4 %) than sPRDM16-K568R cells (54.6 %) (Fig. 3d and e). Similarly, when cell adherence was monitored, as a marker of monocyte differentiation, sPRDM16-WT-THP-1 cells adhered less well than sPRDM16-K568R cells (Fig. 3f). Meanwhile, sPRDM16 SUMOylation was decreased in sPRDM16-WT-THP-1 cells after incubation with PMA (Fig. 3g).

These results suggest that mutation of sPRDM16 SUMOylation site at K568 reduced the capacity of sPRDM16 to induce proliferation and inhibit differentiation of AML cells, suggesting that K568 SUMOylation of sPRDM16 played an important role in the pathogenesis of AML.

### SUMOylation of sPRDM16 enhances the engraftment of systemic THP-1 transplantation leukemia in NOD.CB17-Prkdc^scid^/J (NOD/SCID) mice

**Fig. 4:**
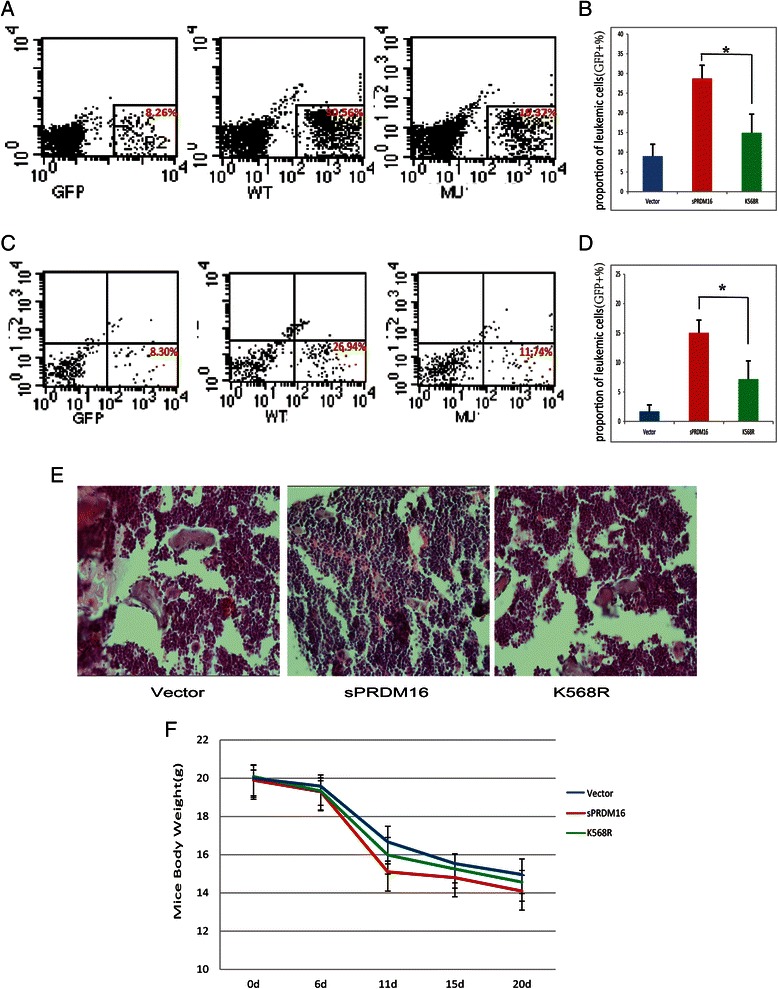
SUMOylation of sPRDM16 enhanced the engraftment of systemic THP-1 transplantation leukemia in NOD.CB17-Prkdc^scid^/J (NOD/SCID) mice. Sub-lethally irradiated mice were inoculated via the tail vein with 1 × 10^7^ GFP-labeled THP-1 cells stably infected with Lenti-Vector, sPRDM16–WT or sPRDM16–K568R. Fluorescent grafts were monitored by flow cytometry. All mice were sacrificed and subjected to subsequent test 30 days post-transplantation of THP-1 cells. **a** FACS blots of leukemic cells (GFP+) in the femur bone marrow in mice inoculated with Lenti-Vector, sPRDM16–WT or sPRDM16–K568R THP-1 cells 30 days post-transplantation. The dot plots showed SSC (y axis) versus GFP intensity (x axis). **b** Bar graphs summarized the percentage of leukemic cell (GFP+) in the bone marrow per femur over whole population (*n* = 5-6). **c** Dot blots indicated leukemic cells (GFP+) in mice peripheral blood 30 days post-transplantation of Lenti-Vector, sPRDM16–WT or sPRDM16–K568R THP-1 cells. The dot plots showed SSC (y axis) versus GFP intensity (x axis). **d** Percentages of leukemic cells (GFP+) in mice peripheral blood over whole population (*n* = 5-6). **e** H&E staining of marrow cavity of femur section from the mice transplanted with THP-1 cells stably infected with Lenti-Vector, sPRDM16–WT or sPRDM16–K568R. **f** Mean animal weight

To confirm our observations *in vivo*, we transplanted sub-lethally irradiated mice with 1 × 10^7^ GFP-labeled THP-1 cells stably infected with Lenti-Vector, sPRDM16–WT or sPRDM16–K568R. The fluorescent disseminated leukemia grafts were monitored by flow cytometry for 30 days post-transplantation. As shown in Figs. 4a and b, in bone marrow fractions, the frequency of GFP + cells in the Lenti-Vector-transplanted group was lower than in either sPRDM16-WT-transplanted or sPRDM16-K568R-transplanted animals. However, the frequency of GFP + cells in the sPRDM16-WT-transplanted groups was higher than in the sPRDM16-K568R-transplanted group. Similar results were observed in analysis of mouse peripheral blood (Fig. 4c and d). To investigate the engraftment of leukemic cells in bone marrow, we subsequently sectioned the bone marrow tissues and conducted hematoxylin and eosin (H&E) staining (Fig. 4e). We also continuously monitored the body weight of mice. Consistent with the flow cytometry results, sPRDM16-WT-transplanted animals lost more weight than sPRDM16-K568R-transplanted and the Lenti-Vector-transplanted animals (Fig. 4f). But the difference was not statistically significant. Taken together, these results indicate that sPRDM16 SUMOylation enhanced engraftment of systemic THP-1 transplantation leukemia in NOD.CB17-Prkdc^scid^/J (NOD/SCID) mice, suggesting that mutation of sPRDM16 K568R partially attenuates the progression of AML *in vivo*.

### Differentiation-related genes induced by PMA are differentially expressed between THP-1 cells stably expressing sPRDM16-WT and sPRDM16–K568R

**Fig. 5:**
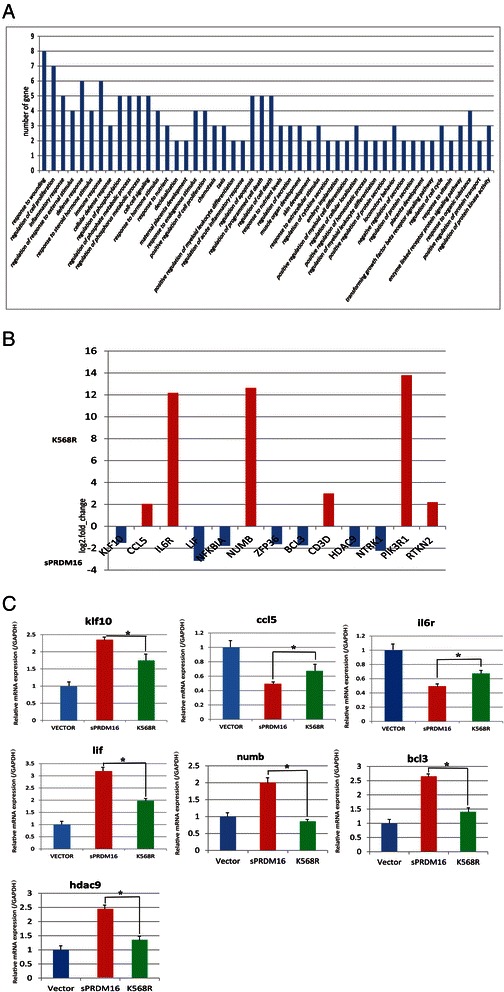
Differentially expressed genes in PMA-induced gene differentiation in THP-1 cells stably expressing sPRDM16-WT and sPRDM16–K568R. **a** Functional annotation clustering (GO) of genes that were associated with SUMOylation of sPRDM16 in sPRDM16-WT and sPRDM16–K568R-THP-1 cells following incubation with PMA (3 nM/24 h; Analyzed by DAVID, grouped based on the biological process of level 1). **b** Differential gene expression between sPRDM16-WT and sPRDM16–K568R-THP-1 cells in mRNA-sequencing. **c** Expression of differentiation-related genes induced by PMA in THP-1 cells stably expressing sPRDM16-WT and sPRDM16–K568R (including control THP-1 cells with Lenti-Vector). KLF10, CCL5, IL6R, LIF, NUMB, BCL3 and HDAC9 was measured in Lenti-Vector, sPRDM16-WT and sPRDM16-K568R cells by Q-PCR. Data are presented as the △Ct between the gene of interest and the GAPDH levels expressed in the same sPRDM16-WT-transduced sample or sPRDM16-K568R-transduced sample, relative to the △Ct observed in the control sample. Mean from three independent experiments are depicted with S.D.

To further investigate the physiological significance of sPRDM16 SUMOylation, we conducted high-throughput sequencing of mRNA (mRNA-seq) with THP-1 cells expressing sPRDM16-WT and sPRDM16-K568R. We analyzed the impact of sPRDM16 SUMOylation global gene expression induced in leukemia cell differentiation using gene ontology. From 10 million 50 bp single-end reads, we identified 237 genes the expression of which differed significantly between sPRDM16-WT-THP-1 and sPRDM16-K568R-THP-1 cells after incubation with PMA (>1.5-fold with adjusted *P* < 0.05). Based on gene ontology results, sPRDM16 SUMOylation significantly affected the expression of cancer-related genes, including genes implicated in wound response, cell proliferation, chemotaxis, differentiation, and cell cycle progression (Fig. 5a). The findings suggest that SUMOylation of sPRDM16 was involved in cancer cell proliferation and differentiation. Such functional enrichment is not surprising as sPRDM16 is a transcriptional regulator of proliferation and differentiation of hematopoietic cell and adipose differentiation.

We next summarized two clusters of gene annotation and selected 13 genes involved in hematopoietic cell proliferation and differentiation (Fig. 5b). We subsequently validated the mRNA-seq results by measuring the changes in 7 newly identified targets, which were previously implicated in hematopoietic cell proliferation and differentiation. The selected targets of sPRDM16 SUMOylation were further validated by real-time RT-PCRs. The RT-PCR results were highly correlated with those observed with mRNA-seq (Fig. 5c). These results demonstrated that SUMOylation of sPRDM16 controls the expression of genes related to cancer proliferation and differentiation, suggesting that SUMOylation of sPRDM16 plays an important role in leukemia cell proliferation and differentiation.

## Discussion

In this study, we demonstrated that sPRDM16 promoted the proliferation and enhanced the self-renewal capacity, of THP-1, while inhibiting differentiation of these AML cells. We further confirmed that K568 is a bona fide sPRDM16 SUMOylation site. Accordingly, mutation of sPRDM16 SUMOylation site K568 partially abolished the influence of sPRDM16 on proliferation and differentiation of AML *in vitro* and *in vivo*. Furthermore, THP-1 cells overexpressing sPRDM16-K568R mutant displayed distinct a gene expression profile from wild type sPRDM16 following incubation with PMA. Our findings suggest that K568 SUMOylation plays an important role in the pathogenesis of AML.

PRDM16 has previously been reported to be involved in myeloid and lymphoid malignancies, and to play a role in the regulation of hematopoietic [12], neuronal stem cell growth [13], and differentiation of adipose tissue [14, 17, 26]. It is widely believed that sPRDM16 is an oncogene [27, 28]. Further, it is well documented that sPRDM16 promotes AML progression by regulating gene transcription through direct DNA binding and/or interaction with transcriptional co-factors and chromatin modifiers [29]. Accumulating clinical evidence indicates that deregulation of sPRDM16 is closely associated with abnormal AML phenotypes [9, 10, 28, 30], implicating sPRDM16 in AML pathogenesis. Consistent with these findings, we found that sPRDM16 promoted proliferation and enhanced self-renewal capacity, while inhibiting differentiation of THP-1 AML cells.

In addition, increasing evidence suggest that SUMOylation may have a major role in the evolution of the hematopoietic system and AML. Some studies have indicated that SUMO is an integral component of chromatin and regulates specific transcriptional programs [31]. Recently, Guillaume Bossis *et al.* reported that an important role of SUMOylation is to regulate the expression of specific genes involved in AML cell response to chemotherapeutic drugs. and inhibition of the SUMO pathway reduces AML cell growth in xenograft mice [6]. The transcriptional activity of sPRDM3, a member of the PR domain family, is negatively regulated by SUMO1 in acute promyelocytic leukemia (APL) [32]. Using various functional assays to compare overexpression of PRDM16-WT or sPRDM16-K568R in THP-1 cells, we demonstrated that sPRDM16 SUMOylation contributed to progression of AML. Furthermore, a SUMOylation mutant of sPRDM16 attenuated its ability to facilitate tumor growth and suppress the differentiation of THP-1 cells *in vitro*. Animal systemic leukemia transplantation models further indicated that sPRDM16 SUMOylation may be a risk factor for leukemia *in vivo*.

Despite the established role of sPRDM16 in leukemia development, the molecular mechanisms underlying sPRDM16 SUMOylation-mediated progression of AML remain elusive. In particular, very few downstream target genes of sPRDM16 have been identified. Our analysis of stable THP-1 cell lines generated by polyclonal lentiviral infection with sPRDM16–WT or sPRDM16–K568R revealed a distinct gene expression profile. mRNA-sequence data indicated no significant difference in gene expression between sPRDM16-WT and sPRDM16-K568R-THP-1 cells in the absence of PMA. Interestingly, following induction of differentiation by PMA, we found that 237 genes were differently expressed between sPRDM16–WT and sPRDM16–K568R-THP-1 cells. Consequently, we confirmed that KLF10, BCL3, HDAC9, CCL5, IL6R, LIF and NUMB, all of which are closely related to differentiation in hematopoietic and leukemic cells [33–37], are downstream targets of sPRDM16, and are influenced by SUMOylation. KLF10 is a transcription factor that regulates differentiation of bone marrow-derived macrophages [33, 38]. BCL3 plays a critical role in targeting the differentiation of myeloid progenitors [34]. The chemokine CCL5 induces selective migration of monocytes and drives their differentiation [36]. Numb plays critical roles in cell fate determination as an evolutionary conserved protein [37, 39]. These results indicate that SUMOylation of sPRDM16 is an important mechanism by which sPRDM16 promotes the growth and proliferation of hematopoietic progenitors and leukemic cells.

## Conclusions

In summary, we have provided evidence that sPRDM16 SUMOylation plays an important role during AML progression by promoting the growth and inhibiting differentiation of AML cells *in vitro* and *in vivo*. We further identified significant changes in downstream genes related to SUMOylation of sPRDM16 during AML differentiation. As sPRDM16 is frequently overexpressed in a variety of leukemias, sPRDM16 SUMOylation may be an important regulator of sPRDM16 in leukemia development.
